# Push and pull factors associated with the consumption of women’s professional basketball games: A canonical correlation analysis

**DOI:** 10.3389/fpsyg.2022.806305

**Published:** 2022-09-06

**Authors:** Sophia D. Min, James J. Zhang, Kevin K. Byon

**Affiliations:** ^1^Department of Kinesiology, University of New Hampshire, Durham, NH, United States; ^2^Department of Kinesiology, University of Georgia, Athens, GA, United States; ^3^School of Public Health, Indiana University, Bloomington, IN, United States

**Keywords:** WNBA, women sports, push and pull factors, fan motives, market demand, sport consumer behavior

## Abstract

The purpose of this study was to empirically investigate the interrelationships between push and pull factors associated with the consumption of women’s professional basketball games. Multiple factors pertaining to sport consumers’ internal needs, identified as “push” factors, contain various intangible socio-psychological motivations representing an individual’s intrinsic desires that drive consumers toward certain goal-driven behaviors. On the other hand, “pull” factors, related to the supply side, refer to the different aspects of sport products the management of sport teams provides. It is imperative to obtain a better understanding of the push–pull interaction so that sport marketers can design their products to satisfy spectators’ expectations with different needs. Spectators (*N* = 628) attending WNBA games responded to an on-site survey. CFA was conducted to ensure the psychometric properties of the scales, which showed that the overall model fit the data well. A canonical correlation analysis was performed, and two significant functions were revealed by the dimension reduction analysis. The first function [*F*(40,2,683) = 4.49, *p* < 0.001]: I-Want-Everything-Consumer suggests that the market segment comprises individuals with multiple needs (ranged from 0.55 to 0.85) and expectations (ranged from 0.55 to 0.89), both of which need to be met simultaneously. Thus, sports marketers can satisfy WNBA consumers’ needs by enhancing the quality of tangible pull factors. The second function [*F*(28,2,222) = 2.38, *p* < 0.001]: Achievement-Seekers revealed that the consumers motivated by vicarious achievement (–0.59) expect game promotion (–0.55) rather than the quality of the opposing team (0.42), indicating that sport marketers should provide tailored promotional strategies to satisfy this segment of consumers. Specifically, the findings of this study can be used to segment consumers based upon fan motives (i.e., push factors) and position products accordingly by managing the controllable aspects of sport products (i.e., pull factors). This study provides empirical evidence of the relationship between WNBA consumers’ multiple needs and attributes associated with the WNBA core product.

## Introduction

Spectator sports continue to grow in popularity and the impact on the U.S. economy. According to Gallup’s sports fan data, 60% of American adults describe themselves as sport fans, and this trend has remained stable since 2000 (Jones, 2015). As stated in the 2021 IBIS World Industry Report (Ristoff, 2021), the U.S. spectator sports industry generated $38 billion in revenue in 2019, and annual growth of 5% is expected from 2021 to 2026. However, only four prime sports leagues —the National Hockey League (NHL), the National Football League (NFL), the National Basketball Association (NBA), and the Major League Baseball (MLB)—generate the vast majority of the U.S. spectator sports revenue. Female professional sports, including the most successful league, the Women’s National Basketball Association (WNBA), and other male professional and semiprofessional sports leagues, share only the remaining small portion of the total revenue that all U.S. spectator sports generate. It is evident that professional women’s sports have not received less recognition from consumers or media coverage and sponsorships.

Although the WNBA is considered the most popular female professional sport league in the U.S. (Johnson et al., 2020), its continued survival and success depend largely on the financial support and reputation of its founder and male counterpart—the NBA (Sandomir, 2016). However, how long the NBA can provide vital financial support remains unknown. Consequently, it may be challenging for the WNBA to obtain sufficient financial support for its continued success. Therefore, the WNBA must develop its marketing and operational strategies to foster consumer interest and ultimately create a loyal fan base that can lead subsequently to increased game attendance and sales of broadcasting and other media rights, which are the primary revenue sources for all professional sport teams (Trail, 2019; Pegoraro et al., 2021). Thus, further research is needed urgently to understand consumers of professional women’s sports so that both the success and survival of the WNBA can be achieved.

With the goal of helping professional women’s sport leagues, particularly the WNBA, become financially independent entities that can enjoy their operational success and commercial viability, this study was designed to examine the interrelation between the supply and demand sides of professional women sports’ consumers. Multiple factors related to their internal needs have been identified. These internal factors are referred to as “push” factors. They include various intangible socio-psychological motivations representing an individual’s intrinsic desires that can subsequently motivate consumers to engage in certain goal-driven behaviors, such as attending sporting events, to fulfill their internal needs. These internal needs can include such sport spectators’ need for an escape from their daily routines, the excitement of intensely competitive games, and vicarious achievement provided by their team’s victory (Wann, 1995; Kahle et al., 1996; Funk et al., 2001; Gladden and Funk, 2001; Mahony et al., 2002).

On the other hand, pull factors related to the supply side refer to the different aspects of sport products that the management of sporting events can provide. These product-related factors include a variety of tangible attractions (e.g., sport events themselves and service quality) that sport service providers can use to “pull” individual consumers to their products. In the field of sport marketing, much evidence has been found to indicate that sport consumers’ evaluations of a variety of pull factors, such as the location of sporting events, the win/loss record of the home team as well as the opposing team, and/or the facilities where sport competitions take place significantly determine their decision to attend sport events significantly (Zhang and Byon, 2017).

Although several factors related to either pull or push aspects of sport events have been documented in sport marketing literature, the extant research has predominantly focused on the effects of push or pull factors alone on sport consumer behaviors rather than examining the interrelation between the factors. Hence, the question, “Based upon what sport events have to offer, what ways can be used to satisfy consumers’ needs?” remains unanswered. Consequently, sports entities (e.g., women’s professional sports) have been unable to create and design product features based upon their products to attract consumers in various market segments with different needs. In brief, this study examines the interrelation between what is needed to satisfy consumers’ internal needs and what women’s professional sports can offer to satisfy those needs. Studies published in mainstream marketing and tourism have stressed the significance of considering the interrelation between push and pull factors (e.g., Kirkwood, 2009; Whyte, 2017), although related information is lacking in sport marketing. This study intended to fill this gap by empirically assessing the interrelation between push and pull factors. The results of this study are expected to contribute to further understanding of sport consumer behavior consuming U.S. professional women’s basketball events.

## Review of literature

### Push factors

Researchers have employed different approaches to increase the understanding of push factors, which are consumer motives that have been defined as “… drives, urges, wishes, or desires which initiate the sequence of events known as ‘behavior”’ (Bayton, 1958, p. 39). Hawkins et al. (2004) described a motive further as “…the energizing force that activates behavior and provides purpose and direction for that behavior” (p. 354). To this effect, sport spectatorship is motivated by various forces that satisfy spectators’ underlying needs and wants.

Several theories relevant to analyzing sport participation and spectatorship have been proposed to study the socio-motivations of sports consumption behavior. Specifically, these sport motivation theories can be organized into five categories: (1) salubrious effects, (2) stress and stimulation seeking, (3) catharsis and aggression, (4) entertainment, and (5) achievement seeking (Sloan, 1989). In addition to Sloan’s (1989) theoretical framework, another line of research follows Mowen’s (2000) meta-theoretical model of motivation and personality (3 M), which provided a rationale for a hierarchy of four levels of personality traits (i.e., elemental, compound, situational, and surface traits). The 3M model maintains that an individual’s cognition, emotions, and behavior are impacted by the interplay of these traits (Licata et al., 2003). By adopting the 3M model, Ko et al. (2017) developed the hierarchical model of sport consumption (H-MSC) and applied it in the context of sport spectatorship and participation to explain sport consumption decision making.

The existing literature on sport marketing has documented a wide range of push factors (internal motives) that could lead to sport spectatorship. For instance, it was reported that spectators attend intercollegiate football games primarily for three reasons: (1) a desire for a unique self-expressive experience, (2) a desire for group affiliation, and (3) an overall attachment to and love of the game (Kahle et al., 1996). In addition, concerning professional sports events, Mahony et al. (2002) showed that three motivations, spectators’ interests in sport, team(s), and player(s), can predict their sport consumption levels significantly. Further, Funk et al. (2001) explained that the factor of vicarious achievement—the feeling that sport spectators’ experience when their favorite team has success—can also serve as the underlying motivation for sport spectatorship.

Another factor, excitement, is supported by previous research that demonstrated that spectators who watch sporting events as entertainment generally attend events that provide a high level of excitement (Funk et al., 2001). With respect to the factor of supporting women’s opportunities in sport, Mc Donald (2000) argued that spectators are motivated to attend women’s sports events, such as WNBA games because they believe that future equal opportunities for women depend upon their support. Aesthetics, “excellence, beauty, and creativity of athletics performance,” has also been found to be a strong motivational factor for spectators (Smith, 1988, p. 58). Drama was also identified as a factor that significantly influences spectators’ attendance at sporting events (Mahony et al., 1999), as spectators motivated highly by drama are emotionally aroused by very competitive games (Funk et al., 2001).

Identifying various sport fan motives successfully has also led to the development of various sport motive scales. For example, based upon Sloan’s (1989) framework, Milne and McDonald (1999) developed a 12-factor sport fan motivation scale to measure needs: risk-taking, stress reduction, aggression, affiliation, social facilitation, self-esteem, competition, achievement, skill mastery, aesthetics, value development, and self-actualization, which can result in sport consumption, such as online purchases of sporting goods or indications of interest in sport. Wann (1995) created another measure, the Sport Fan Motivation Scale (SFMS), to examine individuals’ motives to engage with sport and other sport-related behaviors. The SFMS consists of 23 items and measures the following factors: eustress, self-esteem, escape, entertainment, economic, aesthetic, group affiliation, and family needs. As a first attempt to measure spectator motivation, the SFMS served as a good foundation for developing other spectator motivation scales with better psychometric properties (e.g., MSSC, Trail and James, 2001).

Trail and James (2001) pointed out certain limitations, such as validity concerns, despite the contribution many existing scales have made. Accordingly, the authors developed the Motivation Scale for Sport Consumption (MSSC), a valid and reliable measurement that improved upon the SFMS and Milne and McDonald (1999) scales. The MSSC includes nine fan motivation factors: achievement, acquisition of knowledge, aesthetics, drama, escape, family, physical attraction, physical skill, and social interaction. To examine the socio-motivational factors related to attendance at professional basketball games, Pease and Zhang (2001) developed the Spectator Motivation Scale (SMS), using Sloan’s (1989) categorization as a guideline. This scale includes fan identification, team image, salubrious attraction, and entertainment value, all of which were found to be associated with sociodemographic variables. Pease and Zhang’s (2001) research underscored the importance of considering spectators’ socio-motivations when marketing professional basketball and their sociodemographics when developing marketing strategies.

Funk et al. (2001) developed the Sport Interest Inventory (SII) to measure Women’s World Cup spectators’ motivation in an attempt to include a wider range of motivation factors. The original SII contained 10 motivational factors: sport interest, vicarious achievement, excitement, team interest, supporting women’s opportunity in sport, aesthetics, socialization, national pride, drama, and player interest. The result of Funk et al.’s (2001) study on the SII showed that interest in the sport of soccer, interest in the team, excitement at matches, support for women’s opportunities in sport, aesthetics, and vicarious achievement explained 35% of the variance in interest, the dependent variable. After further investigation, four other factors were added: entertainment value, family bonding, role model, and wholesome environment (Funk et al., 2001, 2002). Funk et al. (2001) conducted studies with sport consumer focus groups to understand individual motivation factors better and include other motives that had not been examined previously. As a result, the SII was expanded with four additional factors, escape, bonding with friends, sport knowledge, and customer service. As demonstrated by multiple linear regression analysis, 10 motivational factors explained 48% of the variance in consumer support. These were as follows: interest in team, escape, role model, aesthetics, socialization, drama, interest in sport, vicarious achievement, support women’s opportunity, and interest in players. Although this predictive ability of the SII was an improvement over the 35% explained for the 1999 Women’s World Cup (Funk et al., 2001), it still fell short of the 54% variance explained for consumer support at the Nike US Cup (Funk et al., 2002). The SII can be modified easily to test spectator motivations in various sports events (Funk et al., 2001, 2002; Pizzo et al., 2018) and has been adopted widely in team sports worldwide (Funk et al., 2004; Neale and Funk, 2006; Wang et al., 2011). Pizzo et al. (2018) compared spectator motives in esports and traditional sport and found that spectators’ distinct sets of motives across contexts influenced game attendance.

### Pull factors

In addition to identifying fan motives (push factors) that consider the sport consumers’ needs and wants, sport marketing researchers have also studied sport spectatorship based upon various pull factors that have significant influences on spectator consumption behaviors. These factors deserve researchers’ attention in that they represent the core product in spectator sports (Mullin et al., 2007; Zhang, 2015). Prior studies have identified various core attributes of a sporting event to better understand sport spectators’ decision-making process. These core attributes are often referred to as market demand. Zhang et al. (1995) defined market demand as the spectators’ expectations of the core attributes of the game itself. The core product may also include other product extensions, including pregame or half-time shows. Market demand can also be explained as a cluster of pull factors that sport organizations can provide to new and returning customers (Zhang et al., 2003; Byon et al., 2010, 2013; Cianfrone et al., 2015). Thus, market demand variables consist of core service quality factors. These can be considered extrinsic factors that “pull” spectators to sporting events, unlike intrinsic factors of the individuals’ internal motivation (Zhang and Byon, 2017; Qian et al., 2020b).

The concept of market demand has been attributed to Schofield (1983), who proposed the following demand categories: demographic variables, economic variables, game attractiveness, and residual preference. Later, Zhang et al. (1995) advanced the knowledge and understanding of market demand’s effects by synthesizing these demand categories. Existing research has identified many aspects of market demand (i.e., home team, opposing team, scheduling, and promotion) and their effects on sports consumption. For example, both the home team and opposing team have been examined as contributing factors to game attendance. Numerous studies have provided evidence of the home team’s significant influence on game attendance. For example, the home team’s win/loss records, league standing (ranking), presence of superstars, and performance were positively related to attendance at NBA basketball games (Zhang et al., 1995). Further, Zhang et al.’s (1997) study revealed that home team history, reputation, league standing, home team quality, and presence of star players also had a positive relation with game attendance at minor league hockey games. Bird (1982) found a direct relation between game attendance and league standing in soccer games. Other studies have focused on variables related to the opposing team, and its quality, history, league standing, and presence of superstars were found to be related to game attendance (Zhang et al., 1995, 1997; Byon et al., 2013).

A positive relationship has been found between game promotion variables and game consumption (Baade and Tiehen, 1990; Zhang et al., 1995). Previous studies (Zhang et al., 1995, 2003) have reported that attendance at professional sports events is related positively to such promotion activities as advertising and direct mail/notification. A number of studies have also provided evidence of the relationship between various economic variables and game consumption (Bird, 1982; Baade and Tiehen, 1990; Zhang et al., 1997). Bird (1982) and Baade and Tiehen (1990) found that the economic consideration affected game attendance negatively. However, Zhang et al. (1995) reported a positive relation between ticket discounts, group ticket costs, good seats, and attendance at NBA games. Furthermore, schedule convenience has also been shown to be related to game attendance. Specifically, spectators preferred to attend weekend or evening games, indicating a positive relationship between schedule convenience and attendance (Zhang et al., 1998). On the other hand, a negative relationship was found between afternoon games and attendance (Hill et al., 1982).

To test the relation between market demand and the National Basketball League (NBA) sport consumer behaviors, Zhang et al. (1995) developed the Spectator Decision Making Inventory (SDMI), which is commonly used as the major market demand scale in sports. The SDMI considers home team, opposing team, game promotion, and schedule convenience. Their study suggested that these four factors accounted significantly for the variation in when individuals decided to attend the NBA’s basketball games. Braunstein et al. (2005) modified the SDMI further and created the Spectator Decision Making Inventory-Spring Training (SDMI-ST) to study MLB consumers. This scale includes four additional variables: vacation activity, economic consideration, nostalgic sentiment, and love of baseball. An NFL’s expansion team has adapted the general market demand concept to explain their product consumption and team identification (Zhang et al., 2004).

Byon et al. (2010) developed the Scale of Market Demand (SMD) with five dimensions for professional sports: home team, opposing team, game promotion, economic consideration, and schedule convenience. The home team dimension was defined as the spectators’ perception of the home team’s quality, as indicated by the team’s win/loss record, reputation, and league standing. In contrast, the opposing team dimension refers to its performance overall, athletic quality, athletic quality of players overall, history and tradition, standing as a rival, and/or superstar(s). The game promotion dimension combines marketing tools that a sport team uses to attract consumers to sport events or consume its product (Kotler and Armstrong, 1996). These tools may include advertising, direct mail, and sales promotions. The economic consideration dimension concerns the economic issues related to the ticket price, including affordability, choice of seat, and discounts that affect the individual. Finally, the fifth dimension, schedule convenience, is represented by the time and day assigned for a sport game event and includes such concerns as to whether the consumer perceives that the schedule is convenient or not. Byon et al. (2013) examined the structural relation of core service quality (pull factor) and peripheral service quality to the consumption of professional sport spectators and the mediating effect of perceived value. Their analyses found that home team, opposing team, game promotion, game amenities, venue quality, and the perceived value predicted behavior intentions.

### Interrelationships between push and pull factors

Previous literature has emphasized that effective marketing requires understanding push (intrinsic) and pull (extrinsic) factors (Baloglu and Uysal, 1996; Qian et al., 2020a; Wang et al., 2020). Both types of motivation have been widely studied (e.g., McClelland, 1985; Ryan and Deci, 2000, 2017; Amabile and Pratt, 2016). Notably, several streams of the literature suggest the importance of understanding the interrelationship between intrinsic and extrinsic motives (Baloglu and Uysal, 1996; Whyte, 2017). For instance, Cerasoli et al. (2014) *via* a meta-analysis suggested the simultaneous presence of intrinsic and extrinsic motivations could jointly improve behavioral outcomes (e.g., job performance) and called for additional research regarding the combined effect of extrinsic and intrinsic motivations on non-performance outcomes.

Deci and Ryan (1985) proposed the Self-Determination Theory (SDT), a macro-theory composed of six mini-theories, including cognitive evaluation theory, causality orientations theory, organismic integration theory, basic psychological needs theory, goal contents theory, and relational motivation theory, to distinguish between intrinsic motivation and extrinsic motivation. Intrinsic motivations refer to behavioral drivers that are inherently volitional (e.g., fun or exciting activities), whereas extrinsic motivations are behavioral factors that are instrumental (e.g., financial rewards). Specifically, as a part of the SDT, adopting a personality approach, the causality orientations theory prescribes that people can take on either an autonomous orientation (e.g., taking an agentic role in one’s own behaviors), a controlled orientation (e.g., adhering to environmental constraints or obligations), or an impersonal orientation (e.g., believing their decisions do not influence the outcome; Sheldon and Prentice, 2019). In addition, the goal contents theory further distinguishes intrinsic goals from extrinsic goals and explains how they differ in their influence on motivation (Ryan et al., 2013). Specifically, the basic needs theory concerns mainly intrinsic motivation and argues that there are three universal psychological needs: autonomy (e.g., volitional control), competence (e.g., efficacy), and relatedness (e.g., social belongingness) (Ryan and Deci, 2019). The relational motivation theory discusses the role of relatedness, which claims that the need for relatedness drives individuals to pursue relationships and that quality relationships provide both bonds with others and satisfy the needs for autonomy and competence (Ryan and Deci, 2019). The organismic integration theory elaborates the effect of extrinsic motivation on human behaviors (Ryan and Deci, 2019).

Although intrinsic and extrinsic motivation are two distinct factors, they are not mutually exclusive and can influence each other (Amabile, 1993). Specifically, as a part of the SDT, the cognitive evaluation theory prescribes that external motivations could affect intrinsic motivation in that they can support or diminish psychological needs (Reeve, 2012). This last property of the macro SDT proposes that sport marketers must identify extrinsic drivers (e.g., home team quality) that do reinforce or, at least, do not weaken sports consumers’ intrinsic motivations for sports consumption. These six mini theories are the cornerstones of SDT, which explains the relationships between intrinsic and extrinsic motivations^1^.

Furthermore, existing studies published in many fields, such as business and tourism, have found a relation between the two factors. For example, using a canonical approach in their tourism study, Baloglu and Uysal (1996) examined the usefulness of the relation between push and pull factors by assigning participants to identified product bundles to create marketing segments and offered important implications for tourism marketing. Similarly, Whyte (2017) employed the canonical correlation approach to study the relationship between travel motives and destination attributes in cruise tourism. Based on his findings, linking push and pull factors in promotional materials can help cruise lines develop more effective marketing and product offerings. Further, a business study Kirkwood (2009) conducted found that both genders were motivated by push and pull factors combined. The study provided a comparative approach based on gender to advance the push-pull theory.

Research on the interrelationships between push and pull factors in the sport industry is sporadic and lacking. Although current research has used ample pull and push factors jointly or independently to predict various sport consumer behaviors (Zhang and Byon, 2017; Qian et al., 2020a; Wang et al., 2020), the sport management literature has gained limited insights into the interrelation between these factors. Thus, although sport marketers can use fan motives (i.e., push factors) and market demand (i.e., pull factors) to segment their consumer market, understanding the interrelation between the two would allow them to tailor their positioning strategies further to individual consumer segments. Consequently, it is imperative to understand the push-pull interaction better so that sport marketers and researchers can design their products to satisfy spectators with different needs and wants.

To address this gap in the sport-related literature, as well as provide additional insights on the interrelationship between extrinsic and intrinsic in non-performance contexts (e.g., sports spectatorship), this study was designed to investigate the interrelationships between push and pull factors associated with the consumption of women’s professional basketball games. The findings derived from this study are expected to advance the existing understanding of push–pull interrelations in sporting events. They derive marketing implications for women’s professional sports, and also help sport marketers position their products better by managing the aspects of sporting events (i.e., market demand) based on the different needs and wants (fan motives) different consumer segments exhibit.

## Materials and methods

### Research design and participants

A multiple cross-sectional survey design was employed, as the data used in this study were collected from WNBA spectators at six different game events. The purposive, volunteer, and convenience sampling method was used to include only spectators aged 18 or older attending WNBA games during a recent (pre-COVID) season. A total of 647 spectators volunteered to participate in this study. Of the participants, 61.7% were female and ages ranged as follows: 41–50 (24.4%), 31–40 (23.3%), and 22–30 (20.1%). The largest ethnic group represented was African American (57.2%), followed by Caucasian (29.2%), Asian (4%), and Hispanic (2.5%). Concerning marital status, 54.1% were single, and 34.8% were married. Household income levels varied among the participants: less than $14,999 (12.3%); $15,000 to $ 34,999 (20.1%); $35,000 to $49,999 (17.6%); and about 50% with an income of $50,000 or more. More than 62.4% reported having an undergraduate or advanced degree. Of the respondents, 20.9% were season ticket holders; most non-season ticket holders purchased single-game tickets (69.1%). Prior to administering the questionnaire, an approval from the Institutional Review Board for the Protection of Human Participants was obtained.

### Measurement

A survey instrument was formulated based on a thorough literature review, including sections on fan motivation, market demand, and demographic information. A number of scales were chosen based on their measurement properties and relevance to the study.

The SII, one of the scales adapted for this study, was originally developed by Funk et al. (2001) and was re-examined and extended by Funk et al. (2002, 2003). The SII has been tested in various sport contexts, and its scale reliability and validity have been supported in multiple women’s sport settings.

Consequently, the SII was chosen over other scales to examine the WNBA fans’ multifaceted motives. Of the total 18 original motivational factors in the SII scale, eight factors were adopted and modified based on theoretical and practical reasons. A panel of experts confirmed the appropriateness and content validity of these factors in the context of women’s professional basketball events. These selected factors included Escape, Aesthetics, Bonding with Family, Vicarious Achievement, Drama, Socialization, Excitement, and Support of Women’s Sport. All items were measured on a seven-point Likert scale ranging from “Strongly Disagree” (1) to “Strongly Agree” (7).

The 17-item Scale of Market Demand (SMD, Byon et al., 2010) was adopted for the current study because the scale measures professional team sports in general without being specific to any particular sport settings. The validity of the SMD was established through rigorous measurement procedures, including a review of literature, reliability and validity tests, and CFA procedures (Byon et al., 2010). The resolved scale also showed good convergent and discriminant validity and internal consistency (α ranging from 0.80 to 0.91; CR ranging from 0.76 to 0.82). The SMD includes the following five sub-dimensions: Home Team (three items), Opposing Team (five items), Game Promotion (three items), Economic Consideration (three items), and Schedule Convenience (three items). All the items are measured on a five-point Likert-type scale ranging from “Not at All” (1) to “Very Much” (5). Additionally, demographic background information was collected for sample description. Included in the questionnaire were the following variables: gender, age, ethnicity, marital status, occupation, education level, household income, and type of ticket. Questions related to these variables were in multiple-choice format.

### Procedures

Once the preliminary questionnaire was formulated, it was reviewed by a panel of experts for content validity testing. The panel included four sport management professors who are experts in measurement and one sport marketing specialist. The format and context of items were evaluated to determine whether they were appropriate, adequate/representative, and accurate/clear (Zhang et al., 1995). The preliminary questionnaire was modified according to the feedback of the panel of expert members, resulting in improved test format, factor relevance, and wording clarity.

Participants recruited for this study were spectators attending Eastern Conference WNBA events in Atlanta, Georgia. The data collections followed a standardized procedure: (1) approaching spectators meeting the age criterion (18 or older), (2) briefly explaining the purpose of the study, (3) informing the spectators that their participation was entirely voluntary and anonymous, (4) providing the informed consent form upon agreeing to participate, (5) collecting the questionnaire when completed, and (6) expressing appreciation for the individual’s time and participation (Zhang et al., 2004). The questionnaire required approximately 10–15 min to complete. A total of 682 questionnaires were collected. Based on Zhang et al.’s (1996) suggestions, 54 questionnaires with non-sporadic missing values were discarded, resulting in 628 to be included in the following data analyses.

### Data analyses

SPSS 27 was used to calculate descriptive statistics for socio-demographics, market demand, and motivation. Although the scales employed in this study were adopted from the previous studies, the data were subjected to confirmatory factor analysis (CFA) to evaluate the psychometric properties of the focal scales applied in women’s professional basketball events. Mplus was used to conduct CFA for push and pull factors. Several goodness-of-fit measures were employed in the data analysis: chi-square statistic(*x*^2^), normed chi-square (*x^2^/df*), root mean square error of approximation (RMSEA), standardized root mean residual (SRMR), comparative fit index (CFI), and expected cross-validation index (ECVI) (Hair et al., 2005).

Evaluating the model’s overall fit using the goodness-of-fit measures must meet certain criteria. The chi-square statistic is expected to show a non-significant difference between expected and observed covariance matrices. For the normed chi-square, a reasonable fit is indicated by a cut-off value of less than 3.0 (Bollen, 1989). Whereas Brown and Cudeck (1992) reported that any RMSEA value less than 0.05 indicates a close fit, Hu and Bentler (1999) suggested that a close fit value could be less than 0.06. A cut-off value less than 0.10 is considered a good fit for SRMR (Kline, 2005). For CFI, a value greater than 0.90 represents an acceptable fit, while a value larger than 0.95 indicates a close fit.

The following tests were performed to measure the reliability of the scales: internal consistency values (Cronbach’s alpha coefficients), construct reliability (CR), and average variance extracted (AVE). For Cronbach’s alpha coefficients, which examine the correlation among the items measuring a specific subscale, and for CR, a cut-off value of 0.70 is recommended (Fornell and Larcker, 1981; Nunnally and Bernstein, 1994). Fornell and Larcker (1981) suggested that AVE values, which evaluate how well the subscale items collectively explain the underlying construct’s variance, be greater than 0.50 to indicate acceptable composite reliability of the construct. Also, AVE values were employed to evaluate the discriminant validity of the constructs.

A canonical correlation was employed to test the interrelationships between fan motives and market demand. The canonical correlation analysis is performed to determine the correlation between two linear combinations determined by the original variables (one from the set of predictor variables and the other from the set of criterion variables). The largest numbers of dimensions that can be derived from a canonical correlation are determined by the smallest numbers of factors in either the criteria or the predictor variable sets. Derived dimensions are orthogonal to each other. The first correlation between the two variates explains the largest possible covariance, whereas the second correlation accounts for the largest possible residual covariance, and so on. The main goal is to determine the function of specific variables in the multivariate relationship (Lattin et al., 2003). The standardized canonical function coefficient (i.e., canonical weight) and the canonical structure loading (i.e., canonical loading) extracted for each variable indicate the relative importance of the variable in each set of variables (Stevens, 2009).

## Results

### Confirmatory factor analysis

A CFA was conducted to determine the goodness-of-fit indices of the eight motivation factors with 40 items (Hair et al., 2005). Goodness-of-fit indices revealed that the eight-factor measurement model did fit the data well (Table 1). The results suggest the motive scale fit the data well (*x^2^/df* = 1997.475/712 = 2.81, RMSEA = 0.054, CFI = 0.905 and SRMR = 0.053). Cronbach’s alpha coefficients for motivation factors ranged from 0.82 for Drama to 0.93 for Vicarious Achievement and suggest acceptable internal consistency based on the suggested cut-off value of 0.70 (Nunnally and Bernstein, 1994). In addition, good construct reliability was indicated by the AVE values ranging from 0.50 for Drama to 0.73 Vicarious Achievement.

**TABLE 1:** Summary results for measurement model of push factor.

Construct and items	CR	λ	AVE
**Escape/Diversion (five items)**	0.87		0.58
The WNBA game provides me with a distraction from my daily life.		0.823	
The WNBA game is a break-away from my routine activities.		0.570	
I could get away from the tension in my life by attending a WNBA game.		0.819	
The WNBA game event provides me with an escape from my day-to-day routine.		0.842	
WNBA games allow me to forget about my problems.		0.735	
**Drama (five items)**	0.82		0.50
I like WNBA games where the outcome is uncertain.		0.739	
A close game between two teams is more enjoyable than a blowout.		0.493	
The uncertainty of a close WNBA game attracts me.		0.836	
The possibility that the outcome of a WNBA game is not decided until the very end.		0.608	
The dramatic turn of events that can take place in a WNBA game.		0.758	
**Aesthetics/Performance (five items)**	0.87		0.58
There is beauty inherent in the WNBA game.		0.728	
I am attracted to the natural elegance of the WNBA game.		0.735	
I appreciate the gracefulness associated with the WNBA game.		0.751	
The style of play of the WNBA provides me with an enjoyable form of entertainment.		0.785	
I like women’s professional basketball games because their style of play emphasizes strategy and the traditional aspects of the game.		0.794	
**Vicarious achievement (five items)**	0.93		0.73
I feel like I have won when the WNBA team wins.		0.831	
The team’s successes are my successes and its losses are my losses.		0.627	
I feel a personal sense of victory when the WNBA team wins.		0.934	
I feel a sense of accomplishment when the WNBA team wins.		0.940	
I become exhilarated when the WNBA team wins.		0.889	
**Social (five items)**	0.85		0.56.
I enjoy the opportunity to interact with other people at the WNBA games.		0.854	
I like the possibility of talking with other people at the WNBA games.		0.840	
I make good use of the chance of socializing with others at the WNBA games.		0.818	
I attend a WNBA game usually due to a friend’s invitation/suggestion.		0.334	
WNBA games have given me a chance to meet other people with similar interests to mine.		0.753	
**Excitement (five items)**	0.92		0.70
I enjoy the excitement surrounding a WNBA game.		0.872	
I find the WNBA games very exciting.		0.863	
I enjoy the high level of excitement during the WNBA games.		0.826	
I enjoy the excitement associated with the WNBA games.		0.837	
Watching WNBA games makes me excited.		0.784	
**Bonding with family/Significant other(s)**	0.90		0.66
Attending WNBA games gives me a chance to bond with my family/significant other(s).		0.764	
I enjoy sharing the experience of attending a WNBA game with family members/significant other(s).		0.648	
An important reason I attend WNBA games is to spend quality time with my family/significant other(s).		0.904	
I attend a WNBA game to enjoy time with my family/significant other(s).		0.947	
I attend a WNBA game to bond with my family/significant other(s).		0.748	
**Supporting women’s opportunity**	0.92		0.69
I attend WNBA games because I think it is important to support women’s sports.		0.834	
My support for the WNBA team is a reflection of my support for women’s sports.		0.864	
Attending WNBA games demonstrates my support for women’s sports in general.		0.804	
Attending WNBA games gives me an opportunity to support women’s sports.		0.799	
I attend WNBA games to cheer for women’s sports.		0.838	

The 17-item measuring market demand was submitted to CFA based on maximum likelihood estimation (Hair et al., 2005). The results of CFA suggested the model fit the data well (*x^2^/df* = 249.605/109 = 2.29, RMSEA = 0.045, CFI = 0.969, SRMR = 0.037), and all item loadings were higher than the 0.707 threshold (Anderson and Gerbing, 1988). The AVE values for all factors were greater than 0.60, with the lowest for Economic Consideration (AVE = 0.64) and the highest (AVE = 0.75) for Schedule Convenience. In addition, CR and Cronbach’s alpha were calculated to further ensure the reliability of the five market demand factors and their respective indicators. The internal consistency measures for all the factors were greater than 0.80, with the lowest for Economic Consideration (Cronbach’s α = 0.84) and highest for Opposing Team (Cronbach’s α = 0.93). With regard to CR, Opposing Team (0.93) had the highest construct reliability, followed by Schedule Convenience (0.90), Home Team (0.88), Game Promotion (0.85), and Economic Consideration (0.84). Table 2 summarizes the psychometric properties of the 17-item SMD.

**TABLE 2:** Summary for measurement model of pull factor.

Construct and items	CR	λ	AVE
**Opposing team (five items)**	0.93		0.71
Opposing team’s overall performance		0.765	
Opposing team reputation		0.877	
Overall quality of opposing team players		0.865	
Quality of opposing team		0.858	
Opposing team exciting play		0.856	
**Home team (three items)**	0.88		0.71
Home team win/loss record		0.805	
Home team reputation		0.846	
Home team league standing		0.883	
**Game promotion (three items)**	0.85		0.65
Advertising		0.746	
Direct mail and notification		0.807	
Sale promotions		0.859	
**Economic consideration (three items)**	0.84		0.64
Total cost for a game event		0.787	
Ticket affordability		0.864	
Ticket discount		0.743	
**Schedule convenience (three items)**	0.90		0.75
Game time of the day		0.851	
Convenient game schedule		0.893	
Day of the week		0.858	

After ensuring the psychometric properties of the two scales, the overall measurement model fit was tested. Based on the result, the overall model did fit the data well (*x^2^/df* = 3203.476/1461 = 2.19, RMSEA = 0.044, CFI = 0.916 and SRMR = 0.044). Of the 57 items, 51 had loadings above 0.707, a high and conservative criterion (Anderson and Gerbing, 1988). In addition, it has been suggested that an indicator loading of 0.707 or greater indicates the pattern coefficient achieves meaningful significance (Hair et al., 2005). Therefore, this measurement model excluded six items (e.g., “I attend a WNBA game usually due to a friend’s invitation/suggestion”) based on this criterion. The final measurement model consisted of 13 factors with 51 items.

### Canonical correlation analysis

A canonical correlation analysis was conducted to test the interrelationship between push and pull factors. Stevens (2009) suggested that instead of using individual items to investigate the relationships between predictor and criteria variables, factor scores increased the subject-to-variable ratio and improved our confidence in interpreting the results derived from canonical correlation. The final canonical correlation was conducted on five factors in the predictor set and eight criteria set with 628 subjects. Therefore, a 48-to-1 subject-to-variable ratio was achieved, allowing the two largest canonical correlations to be confidently interpreted (Stevens, 2009).

The canonical correlation analysis findings showed five functions with canonical correlations ranging from 0.07 to 0.40. Across the functions, the full model was statistically significant: Wilks’ λ = 0.75, *F*(40,2,683) = 4.49, *p* < 0.001). As Wilks’ λ represents the variance unexplained by the model, for the set of canonical functions, the full model effect size could be determined by (1– λ). Hence, 25% variance was shared between the independent and dependent variates. The dimension reduction analysis revealed two significant functions. To be more precise, Function 1 [*F*(40,2,683) = 4.49, *p* < 0.001] and Function 2 [*F*(28,2,222) = 2.38, *p* < 0.001] were statistically significant and were considered noteworthy for this study, whereas the remaining three functions failed to reach statistical significance. The standardized canonical function coefficients and structure coefficients for Functions 1 and 2 are presented in Table 3. Also provided are the squared structure coefficients and the communalities (*h*^2^) for each variable of the two functions. For Function 1, all variables exceeded the criterion of standardized canonical function coefficients and structure coefficients of 0.40 or greater (Stevens, 2009).

**TABLE 3:** Descriptive statistics and canonical solution for push factor predicting push factor for functions 1 and 2.

	Min	Max	Mean	*SD*	Skewness	Kurtosis	Function 1			Function 2			
Variable							Coef	*r*_*s*_	*r*_*s*_^2^(%)	Coef	*r*_*s*_	*r*_*s*_^2^(%)	*h*^2^ (%)
**Predictor set**													
Escape	1	7	5.20	1.45	–0.80	0.76	–0.19	0.56	30.85	0.06	–0.11	1.15	32.00
Drama	1	7	5.63	1.19	–1.07	1.272	0.47	0.86	75.20	0.55	0.27	7.40	82.61
Aesthetics	1	7	5.70	1.14	–1.21	1.775	0.43	0.89	80.29	0.12	0.03	0.07	80.36
Vicarious Achievement	1	7	5.22	1.53	–0.80	0.347	0.22	0.72	53.03	–1.12	–0.59	34.73	87.76
Social	1	7	5.33	1.32	–0.81	0.11	0.80	0.69	48.97	–0.40	–0.38	15.63	63.61
Excitement	1	7	5.90	1.12	–1.49	2.99	–0.19	0.77	60.71	0.51	0.07	0.48	61.20
Bonding with family	1	7	5.51	1.49	–1.17	0.94	0.20	0.56	31.95	0.14	0.05	0.21	32.17
Supporting women’s opportunity	1	7	6.14	1.10‘	–1.82	4.02	0.21	0.72	49.20	–0.003	0.04	0.14	49.34
*R*_*c*_^2^									15.76			6.65	
**Criterion set**													
Opposing team	1	5	3.74	0.93	–0.94	1.14	0.19	0.74	55.44	0.86	0.42	19.92	73.35
Home team	1	5	3.81	0.97	–0.85	0.62	0.26	0.77	59.60	0.05	0.16	2.53	62.12
Game promotion	1	5	3.21	1.14	–0.33	–0.56	0.45	0.78	60.82	–0.93	–0.55	30.18	90.99
Economic consideration	1	5	3.66	1.02	–0.68	0.17	–0.29	0.55	28.76	–0.36	–0.26	7.01	35.77
Schedule convenience	1	5	3.71	1.00	–0.75	0.38	0.56	0.85	70.79	0.29	0.08	0.67	71.46

Structural coefficients (*r_s_*) greater than |0.40| are underlined. Community coefficients (*h*^2^) greater than 50% are underlined.

Coef, standardized canonical function coefficient (i.e., canonical weight); *r_s_*, structure coefficient (i.e., canonical loading); *r_s_*^2^, squared structure coefficient; *h*^2^, communality coefficient.

In Function 1, all variables in the criteria variate (i.e., market demand) had loadings exceeding 0.50 and resulted in a highly shared variance (0.85), indicating a high degree of intercorrelation among the variables, suggesting that all factors were representative of Function 1. Although all variables contributing to the criterion variate were positive, Schedule Convenience (0.85), Game Promotion (0.78), Home Team (0.77), Opposing Team (0.75), and Economic Consideration (0.55) can be considered as the primary contributors, as supported by the high squared structure coefficients and *h*^2^.

**FIGURE 1:**
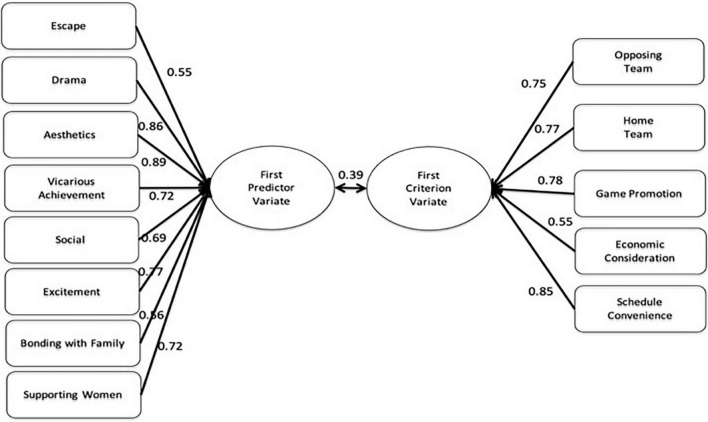
Canonical functions with primary contributing factors canonical functions 1.

Canonical loadings for the first predictor variate had a much wider range (0.55 to 0.89), with all positive loadings. The primary contributors were Aesthetics (0.89), Drama (0.86), Excitement (0.77), Vicarious Achievement (0.72), Supporting Women’s Sports (0.72), Social (0.69), Bonding with Family (0.56), and Escape (0.55) to the predictor variate. The high squared structure coefficients and *h*^2^ supported this conclusion. All the structure coefficients were positive, indicating that all were positively related. These findings support the expected significant relationships between fan motives and market demand. The findings of Function 1 represented the first way of the relationship between the internal and external motivational contributors (I- Want-Everything) (see Figure 1).

**FIGURE 2:**
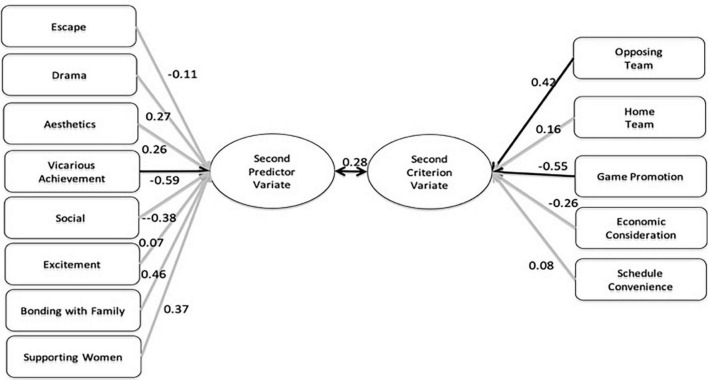
Canonical functions with primary contributing factors canonical functions 2.

Also shown in Table 3 are standardized canonical function coefficients and structure coefficients for Function 2. Of note, Function 2 explains the deviance of Function 1 and thus reflects a second process independent of the first way represented by Function 1 (Achievement Seeker). Based on the results of Function 2, criterion variables of relevance were Opposing Team (0.42) and Game Promotion (–0.55), whereas the major contributor in the predictor variate was Vicarious Achievement (–0.59). Generally, these results supported the expected relationship between Vicarious Achievement and Game Promotion (see Figure 2).

## Discussion

The purpose of this study was to investigate the interactions between push and pull factors empirically, which can contribute to understanding sport consumer behavior and market segments. Specifically, the findings of this study can provide segmentation and position strategies that sport marketers can use to segment their consumers based upon fan motives (i.e., push factors) and position their products accordingly by managing the controllable aspects of sport products (i.e., pull factors).

### Theoretical contributions

Previous literature has emphasized the importance of understanding motivation. Most prior studies on fan motives have investigated their influence on consumption (e.g., Funk et al., 2001; Mahony et al., 2002), while others (e.g., Trail et al., 2003; James and Ross, 2004) have examined market segmentation with a focus on psychographic motivational factors. Despite these studies’ valuable contributions to motivation, no positioning strategies have been offered based on empirical results.

Market demand is the term Zhang et al. (1995) advanced to refer to spectators’ expectations related to the core attributes of the game itself, which have been explained as a cluster of pull factors that sport organizations can provide to attract new and retain returning customers. These market demand variables consist of core service factors that function as extrinsic factors that attract spectators to sporting events. Hence, as the controllable aspects of sports products, these market demand factors have great potential for sport marketers. Such factors can be used to target sport products to different consumer segments.

In the sport management literature, extant studies have extensively treated push factors (motives) and pull factors (market demands) as two separate constructs and used them to separately predict sports consumption. However, few, if none, studies have tested the relationship between these two constructs, leaving a void in the understanding of sports consumer behaviors such that the relations between the supply side (i.e., pull factors) and demand-side (i.e., push factors) are unclear. Therefore, this study extends previous research by focusing on both the needs that motivate spectators to attend game events and the expectations marketers can control and improve and their interrelations. This study contributes to developing positioning strategies by underscoring the importance of market demand as a powerful tool in satisfying consumers.

Through canonical correlation analysis, this study examined the multivariable relation between pull and push factors. The findings revealed two significant functions, referred to as the “I-Want-Everything-Consumer” and “Achievement Seekers.” As the name indicates, the first I-Want-Everything-Consumer function relates to all eight fan motives—aesthetics, social interaction, drama, vicarious achievement, excitement, supporting women’s sports, escape, and bonding with family—which loaded relatively highly (λ ranged from 0.55 to 0.89) on the predictor variable. With respect to the criterion variable, opposing team, home team, game promotion, economic consideration, and schedule convenience, all had relatively high loadings (λ ranged from 0.55 to 0.85) with the same sign and thus were correlated positively with each other.

Overall, the first function suggests that rather than being motivated by only one need, multiple needs may determine sport consumption simultaneously. With respect to consumers who have multiple needs to be satisfied, the I-Want-Everything-Consumer function suggests that satisfying consumers who are motivated by aesthetics, social interaction, drama, vicarious achievement, excitement, supporting women’s sports, escape, and bonding with family can be achieved by meeting all of their expectations, such as quality of the opposing and home teams, game promotion, economic consideration, and schedule convenience. Accordingly, these findings highlight the importance of recognizing these two market segments composed of individuals with multiple needs and expectations that must be met simultaneously. This finding implies the significance of the relationship between motivation (push factor) and market demand (pull factor) that leads to successful marketing strategies.

In the second function, vicarious achievement had a relatively high loading (λ = –0.59) on the predictor variable. On the criterion variable side, the opposing team had a negative loading (λ = 0.42), while game promotion had a positive loading (λ = –0.55). Therefore, the opposing team was correlated negatively with vicarious achievement, while the game promotion was correlated positively in Function 2. The differences revealed by the second canonical correlation function should be noted. The results of Function 2 can be interpreted as indicating that the consumers motivated by vicarious achievement expect game promotion rather than the quality of the opposing team. Such consumers who want to experience achievement vicariously through their association with their team would certainly not want the opposing team to be a strong competitor that might have a high probability of defeating the home team during games. On the other hand, game promotional materials, such as giveaways, email notifications, or any other promotions, can enhance vicarious achievement by valuing spectators, showing them appreciation, and making them feel that they are a part of the team.

### Managerial contributions

The findings of this study have implications for sport marketing working in professional women’s basketball organizations. One important implication is that a good understanding of the relationship between consumers’ needs and expectations is necessary for effective sport marketing. This study provides evidence that developing a successful marketing strategy requires recognizing the consumers’ multiple needs and understanding the way products can be improved to meet their expectations. Market demand refers to the core products that are tangible and under marketers’ control. These core products include opposing team, home team, game promotion, economic consideration, and schedule convenience. The two functions derived from the canonical correlation provide useful information for sports marketers to design and position their products by controlling these market demand aspects.

#### Function 1 – I-want-everything-consumer

Function 1 shows that sport marketers need to improve all market demand factors to satisfy consumers with multiple needs. For example, strategies that focus on opposing team and home team quality could be more successful if greater attention is given to scheduling more rival games that create more excitement and developing more star players. For example, interest in ticket sales skyrocketed after the acquisition/transfer of WNBA stars such as Phoenix Mercury’s acquiring Skylar Diggins-Smith, resulting in an 837% increase compared to the previous year. Although marketers can continue designing effective strategies that incorporate a variety of promotional events before, during, and after games, more long-term promotions (e.g., year-round institutional advertising) rather than just *ad hoc* promotion (e.g., in-game amenities).

Currently, the WNBA offers a variety of ticket packages to accommodate its fans, such as family ticket packages that include concessions and merchandise and packages that allow savings and flexibility in game selection. However, other economic strategies should also be considered. Ticket packages that include other hidden costs can be more attractive to fans. For instance, parking packages can be more economical for fans, as most of the WNBA home team arenas are located in downtown metropolitan cities, where parking can be an additional expense that can even exceed the cost of a single ticket. In addition to its current strategies, the WNBA can benefit from other effective ticket strategies.

Effective marketing strategies could also consider the scheduling of games. Currently, the WNBA has a schedule that may be inconvenient for many potential fans (e.g., weekday mornings). Although media schedules partly influence this, WNBA must adopt a better schedule to increase event attendance.

#### Function 2 – Achievement seekers

Function 2 revealed that achievement seekers, motivated by vicarious achievement, value game promotion rather than the opposing team’s quality. The implication is that sport marketers should provide more long-term promotional strategies to satisfy this segment. For example, promotions increase fans’ team identification and build a stronger relationship to enhance their vicarious achievement by appreciating and valuing spectators as part of the team. The WNBA can benefit from providing game promotions, such as more team merchandise giveaways, advance notifications, or special pre/post-season offers and events designed just for fans.

Further, promotional events that encourage fans to wear team apparel to show their fanship can enhance vicarious achievement, increasing their involvement and team identification. Further, as achievement seekers do not expect the opposing team’s quality, teams should avoid promoting rival game events. Instead, promoting their strengths while focusing on their potential chance to win may be a more desirable strategy.

### Limitations and suggestions for future studies

Several limitations in this study should be noted. First, the study provides insight into the interrelation between push and pull factors. However, the conclusions are offered merely as suggestions. It was an exploratory study, and the exploratory techniques employed did not allow consumer motivation or market demand to be tested directly. Although the findings indicate that relations exist between push and pull factors, this study cannot explain the exact relations. The study has limited capacity to be generalized. The data for this study were collected only from research participants who attended the games of one particular WNBA team franchised in a metropolitan region of the Southeastern U.S. Thus, caution should be exercised when interpreting the findings, as the sample may not reflect consumers of other professional leagues or sports as well as nor other types of consumption form such as TV, online, merchandise, etc.

Another limitation is that this study did not consider differences in the level of team identification, involvement, and consumption. Some previous studies have distinguished between die-hard and fair-weather fans and reported that the former are more likely to be loyal and supportive regardless of the team’s performance and pay more attention to core products of sport events, while ticket sales for fair-weather fans may be more affected by the team’s win-loss record. Therefore, future research should recognize these differences (i.e., highly identified vs. lowly identified) among fans and their different expectations to provide more specific segments. In addition, this study examined only the core products for positioning. Other peripheral aspects, such as service quality, which refers to consumers’ assessment of the organization’s service, including quality of the facility, convenience, and environment, should be considered. As service quality is controllable and tangible, it deserves more attention in future studies. Also, as the current study did not consider competitive balance, another determinant of sport fan consumption, future studies can extend the current work by comparing the level of competition in games between teams that are equally matched or those that are not. Lastly, as this is an exploratory study, we did not test for causality of the relationships or perform the invariance analysis by gender or other relevant variables; however, scholars should perform such tests to further our understanding of the nuanced differences and similarities of the relationships between pull and push motive factors among specific segments of sport consumers (e.g., gender).

## Data availability statement

The raw data supporting the conclusions of this article will be made available by the authors, without undue reservation.

## Ethics statement

The studies involving human participants were reviewed and approved by Institutional Review Board, University of Georgia, United States. The patients/participants provided their written informed consent to participate in this study.

## Author contributions

SM was the primary composer of the manuscript and performed the primary data collection and data analysis. JZ provided insight into the conception, design, and execution of the research, and contributed immensely to the overall improvement in the quality of this study by giving critical feedback based on his profound research insight and experience. KB made significant contributions, such as providing critical feedback, advice on data analysis, and suggestions for the initial drafts and revisions, all of which were instrumental in developing and improving this manuscript. All authors contributed to the article and approved the submitted version.

## Footnotes

1 For a detailed review of the six mini-theories, see Reeve (2012).
